# The Long Fox Lecture

**Published:** 1904-12

**Authors:** John Beddoe

**Affiliations:** Formerly Physician to the Bristol Royal Infirmary


### THE LONG FOX LECTURE:

THE FIRST ANNUAL LECTURE ARRANGED BY THE COMMITTEE OF THE LONG FOX MEMORIAL,

DELIVERED IN THE MEDICAL LIBRARY, UNIVERSITY COLLEGE, BRISTOL, ON NOVEMBER 4TH, 1904.

THE RIGHT HON. LEWIS FRY *in the Chair.*

BY

JOHN BEDDOE, M.D., LL.D., F.R.S.,

*Formerly Physician to the Bristol Royal Infirmary.*

---

WHEN made aware that I was to have the honour of delivering the first Long Fox Lecture, I naturally gave some thought to the selection of a suitable subject.

There are sundry lectureships having more or less analogy with this one. Some of these have been founded at least as much with the view of commemorating the founders themselves, of rescuing their names from the common lot of oblivion, as for any object of general utility. And well may we sympathise with such a desire! It is hard for most of us to reconcile ourselves to that common lot; it is hard to say with resignation—

> "Thus shall it chance to me:
> In ages yet to be
> There shall remain no trace on land or sea,
> Nor in the memory of any friend;
> But they and it shall surely have an end."

But more germane to this foundation of ours are the Harveian Lecture of the College of Physicians and the Huxley Lecture of the Anthropological Institute. How differently are these two handled! The Harveian Lecture has become a kind of *tour de force.* The orator feels bound to lay yet another wreath on the shrine of Harvey, already almost hidden from view by previous contributions, and to display his ingenuity in straining facts to fit in with the notion that that undoubtedly great man was, what he was not, another Aristotle or a greater

Crichton. On the other hand, the last Huxley Lecture to which I listened, delivered by an eminent man, and containing much that was new and important, included no mention that I heard of the illustrious name of Huxley; nor did I recognise any allusion even to his work. Both these extremes should surely be avoided. But though such considerations passed through my mind, I had no difficulty in choosing a subject.

This is the inaugural lecture of a series instituted to honour and commemorate an able, a laborious, an unselfish and generous physician and philanthropist, a man who loved his profession and his fellow-creatures; and I have chosen to speak to you of "the ideal physician." I am not careful to define what I mean by the word physician. It is, of course, a misnomer, meaning properly a natural philosopher, or what some people now call by the ugly name of scientist. But the conception has been gradually narrowed in this country, until nowadays in common speech it is confined to those who belong to the College, and practice according to its requirements. No doubt a certain dignity is maintained by the reception of payment in the guise of a fee: the transaction is thus lifted in some measure above the mere commercial level, with its liability to chaffering and haggling. "Let baseborn peasants buy and sell! I gave the cloak to thee," said Syloson to Darius. Less and less can this unpractical view obtain in a country where material civilisation and luxury advance as they do in ours; but the tradition is an honourable and generous one; and we may be permitted to hope that it will not yet altogether perish from among us.

The subject might be envisaged from more than one point of view. I shall endeavour to treat it historically.

The beginnings of medicine are inscrutable: those of the profession are obscure. It is not really an offshoot from the clerical one, as is by many supposed. When anthropologists wish to get as near as may be to the beginnings of institutions, they investigate the customs of contemporary savages. The Australians are not so black as they are painted, nor so primitive as they are called; but they may serve for an example. They know the uses of sundry excellent drugs, and of some good surgical procedures: their treatment of snake-bite, for example, is extremely judicious, and seems to be occasionally successful. But they have no acknowledged mediciners: as Dr. Roth says, these things are common knowledge, however learned. I suspect it is in the hands and brains of the old women of the tribe. The so-called medicine men, wizards or conjurors, who are the first stage of the evolution of the priest, have nothing to do with it; and this seems to be the general rule elsewhere, and at considerably later stages of civilisation. Asa, King of Judah, was blamed because, when in his old age he was afflicted with gout, he consulted the physicians rather than the priests. Where superstitious feeling is strong and science at a discount this antagonism still crops out as it did of yore. In Asia Minor, where I once had a little gratuitous practice among the Moslem, my rivals were the Mullahs, who read the Koran over the sick person with curative effect, much as the Peculiar People, Christian Scientists, Mormons, and the like do among ourselves.

An exaggerated respect for authority is an impediment to the progress of medicine, as it is to that of every art or science; and it is so whether the authority so worshipped is supposed to be religious or scientific. On the other hand, the stability and durability of nations or political powers are greatly favoured by such excessive reverence for authority. This characteristic was strikingly marked in the four countries in which, owing to their physiographical endowments, civilisation was apparently earliest developed to a high stage. These were Egypt, the valleys of the Euphrates and the Tigris, and, somewhat later, India and China. In all of these the progress of medical science appears to have been early arrested; and the practitioners of medicine, accustomed or even compelled *jurare in verbo magistri*, naturally failed to make any further advance. The surpassing excellence of the teacher, in accordance with Emerson's principle of compensation, became an actual obstacle to the progress of his disciples and their posterity. Charaka and Susruta were the Hippokrates and Galen of Indian medicine. Some of their work might be read with instruction even at the present time; and if I had to borrow the portrait of a perfect physician from any single writer or book I could mention, I am by no means sure that I would not choose the Shastres. What can be more important for the physician (and for his patients) than that he should have a good memory and a calm temper? These were put in the forefront of necessary qualifications by the old Hindu sages; and many of their medico-ethical precepts are equally commendable, and would be as useful to the young European as they doubtless were to the young Hindu practitioner. The union of practical observation and training with a thorough knowledge of the Shastres was insisted on; but unhappily, in medicine as in religion, dogma is more tenacious of life than practical morality or right conduct, and in modern Hindu medicine there is not very much that is worthy of respect.[1]

I am unaware whether much is known as to the state of medicine in the latter days of Babylonia; but the Laws of King Hammurabi seem to indicate that in early days it was in low estimation, that surgery was more cultivated than medicine, and that its practitioners were in an inferior and precarious position.

As for Egypt, Herodotus says that every physician applied himself to one disease and not more; or rather, as he subsequently qualifies the statement, some of them were for the eyes, others for the teeth, others for the head, others for internal diorders, and so forth. This excessive specialisation indicates, I think, a stereotyped condition of the whole art, rather than that accumulation of minute knowledge which nowadays leads to a similar division of labour. Probably none of his successors dared venture to improve upon the dicta of the universally-gifted semi-divine Ierhotep. It is a marvellous and a melancholy consideration that with all the anatomical opportunities supplied by their practice of embalming, the Egyptians just missed the discovery of the circulation of the blood.[2] That Darius's surgeons were Egyptians was probably due less to their reputation for extraordinary skill than to the fact that Darius, himself belonging to a military, aristocratic, and comparatively uncivilised race of highlanders, had participated in Cambyses's conquest of the highly-civilised Egyptians.

[1] Wise's *Hindu Medicine*.

[2] Caton's Harveian Lecture, *Lancet*, 1904, i. 1769.

Herodotus tells us that whereas Darius had dislocated his ankle by leaping from his horse during a hunt, these Egyptians were unable to cure him, and indeed rather aggravated his condition by their unskilful procedures. The case is somewhat difficult to understand from Herodotus's story; but the King was ultimately restored to health and his lameness cured by the treatment of Demokedes of Krotona, a Greek surgeon of established reputation. I speak of him now, firstly because he is the first known to us with certainty of a long line of illustrious healers of men; and secondly because he affords the first known instance of professional loyalty, a virtue certainly essential in the ideal physician. For whereas Darius, though not more cruel than the ordinary Oriental despot, had ordered the wretched Egyptians to be impaled as the penalty of their failure, Demokedes intervened, and obtained their pardon from the tyrant. He may, of course, have been actuated by a judicious dread of allowing the creation of an inconvenient precedent; but one prefers to acknowledge the probability of a better motive.

As there were brave men before Agamemnon, so doubtless were there skilful healers before Hippokrates, and even before Demokedes; nevertheless, the coming of Hippokrates might, with more truth than in another famous instance, be compared to that of "the sun in his strength." His contemporaries, men of an average type inferior to that of no earlier or later race, held the same high opinion of him which his writings, fragmentary though they be, have ever since maintained in existence. It would be no exaggeration to say that there were few subjects pertaining to medicine, of which anything was known before the invention of the microscope, of which Hippokrates had not taken thought; and that no physicians, and perhaps few surgeons, have not been better equipped for practice by a perusal of his works. What does he say, then, as to the qualifications of the physician? The student should, he tells us, have sufficient capacity, leisure and means for obtaining instruction in a favourable place, whereby I suppose he meant a place where sufficient material was at the command of competent teachers, as may have been the case at Krotona and at Kos. He must bring to the task industry and perseverance. He must, above all, be *καλος και αγαθος*, fair without and good within. We may take *καλος* in different senses: if we interpret it "handsome, prepossessing in aspect," all here and on this particular occasion will be ready to recognise its applicability; but no doubt Hippokrates intended it to have a wider application. It is true that he did not disdain—what worldly-wise man would?—to consider sometimes what methods of behaviour were conducive to what we among ourselves call success in practice, and how one could most favourably impress his clientry; but this was not the leading object in his mind: he desired above all things that his pupil should become a good physician, a skilful healer, and attain success, if he did attain it, by real desert and legitimate means; that he should cultivate "whatsoever things were lovely and of good report."

Thus in the "Oath," that curious and valuable document, which Adams pronounces pretty certainly authentic, that is, the genuine production of Hippokrates, and which may have been the engagement entered into by his pupils on their reception as such, we find, indeed, stipulations inculcating loyalty to the profession and the teacher, but other and more numerous ones directed to the culture of general as well as of professional rectitude.

The pupil is to reckon him who has taught him this art of medicine equally dear with his parents, to share his substance with him and minister to his necessities if required, to look upon his offspring in the light of brothers, and to teach them this art without fee or stipulation, and to impart the knowledge of it to his own sons and to those of his teachers, and to disciples bound by a stipulation and oath according to the law of medicine, but to none other. He is to give no deadly drug to anyone, whether spontaneously or on request, nor to give anything deleterious to a woman; and to live and practise with purity and holiness. Into whatsoever house he may enter he must enter with the object of benefiting the sick, and he must abstain from every act of mischief, corruption or vice. And whatever he may come to know, whether in connection with his professional practice or otherwise, which ought not to be spoken of abroad, he is not to divulge, but to keep secret. This brief but admirable code has come down to us through more than two thousand years: we still hold to it in substance; and within its limits it is scarcely capable of improvement.

Another important consideration may be included under the word *καλος*. The healer must be himself hale—hale and whole. He needs not to be athletic, but he must be physically sound; and more especially his nervous system and organs of sensation must be healthy, though sensitive. In few if any professions is acuteness of the senses of sight, of hearing and of touch equally essential.

Consider again the first Aphorism, the former part of which is familiar to all of you, though not all of you are aware that we owe it to Hippokrates: "Life is short, and the Art is long; the occasion fleeting;" that is, as Adams interprets it, "the season during which remedies may be successfully applied soon passes"; "experiment is fallacious" or "dangerous," "and decision is difficult." "The physician," he continues, "must not only be prepared to do what is right, that is what is necessary or expedient, but to make the patient, the attendants, and all around co-operate with him in his object."

How deeply this master of his art—for he was its master,—and if the art was very imperfect, as it still is after seventy generations of men have struggled with it, that does not alter the fact that he brought to its study and practice a brain nowise inferior to that of any of the illustrious men who have followed in his path—how deeply, I say, did this great master feel the difficulties and responsibilities attaching to medical practice.

"Who shall decide when doctors disagree?" How often are we reminded by others of this trite saying, always in a somewhat depreciatory sense, as though it were a reproach to the medical profession that such disagreement should be not only possible, but frequent. I once heard a very distinguished judge make use of the expression in that sense. In truth, medicine, dealing as it does largely with the unseen and the conjectural, is a far more difficult subject than law; and yet we are accustomed to see without surprise one legal case after another made the shuttlecock of eminent lawyers, as it passes, or rather wriggles and zigzags from one appeal court to another.

The saying was not in its origin meant to apply to mediciners at all. It was the doctors of the Middle Ages, the holders of degrees in arts, philosophy and divinity, the angelic and seraphic doctors of the church, who wandered in the tangled mazes of metaphysics and theology, and whose very life was in disputation and paradox, who were really pointed at.

It is perhaps to be regretted that this word doctor, properly signifying teacher, and applied to those only who were considered competent to teach their art and mystery, should in England have become the popular appellation of professors of the healing art, of whom at most a minority have any title to it whatever. It is a mistake peculiar, I believe, to English-speaking countries. Leech, meaning a healer, would be preferable, as it is found with that signification in all or almost all Germanic and Keltic languages. Thus Mac-an-leigh—son of a physician or healer—was the true patronymic of David Livingstone, Livingstone being a random attempt at a translation from the Gaelic into a well-known Saxon surname.

The usual controversies and parties in medicine had their origin as far back as the time of Hippokratés: they are indicated rather than debated in his writings, and have come into prominence whenever the science or art of healing has made any notable advance.

Whether and to what extent philosophical or scientific education should precede practical teaching, and whether and to what extent practice and observation outweigh the value of such education, gave rise to as much doubt and division then as now.

Galen gives us what is evidently meant for an estimate or portrait of Hippokrates; it may be somewhat idealised, but, after all, it is the ideal physician whom I desire to portray. "He who esteems opulence," he says, "more than virtue, and who has learned his art in order to amass riches rather than for the good of human kind, cannot even draw near to the object which medicine aims at. . . . It is impossible, in fact, to be covetous of riches, and at the same time worthily to cultivate this noble art; he who attaches himself ardently to the one will certainly neglect the other." "If one could find nowadays," he continues, "a physician so free from sordid considerations that he would not only teach by discourse but demonstrate by his conduct that one should be content with the necessaries of life, with enough of food, drink, and shelter, such a physician would disdain the favours of kings such as Artaxerxes and Perdiccas: he would be ready to treat the latter if he were suffering from a malady which required his help; but rather than remain as his official attendant he would wander to Krannon or Thasos, where his skill was needed by the poor inhabitants. He would leave his disciples to minister to the wants of his fellow citizens, while he himself would think it necessary to travel far and wide in order to verify by experience the theoretical views at which he had arrived, on the nature and relative merits and the influence on health of sites and aspects or exposures, and of potable waters. No covetous or luxurious or sensual man could behave thus," he continues, "the true physician is an ardent lover of labour, a friend of temperance, a disciple of the truth." He "scorns delights and lives laborious days."

Galen, going astray as he sometimes did in the elaboration of verbal logic and the Socratic method of reasoning, maintained that the true mediciner must of necessity be a philosopher, because the possession of the characteristic virtues of a philosopher was essential for the right practice of physic. But apparently he included besides wisdom, justice, temperance, altruism, skill in logic, and what we used to call natural knowledge; and some at least of these, he remarked, could not be learned without a master and without practice. He thought, therefore, that a good general education such as was possible and usual in his days was a necessary preliminary; and in discussing the demerits of the several medical sects then existing he bore particularly hardly on the Empirics, who thought they needed nothing but practical knowledge.

Celsus, however, seems to me to have put the case better, and after briefly stating the arguments on the subject which were relied on by the several sects he summed up with Latin directness. For whereas the knowledge of anatomy was very imperfect, and that of physiology and pathology dubious or non-existent, and the results of vivisection were apt to be deceptive, Celsus briefly commented that nothing contributed more to a rational method of cure than experience; yet that many kinds of knowledge, though not properly nor absolutely essential to the art, were yet helpful to it by quickening the understanding of the practitioner; therefore the contemplation of the nature of things, though by itself it could not make a physician, yet made a man more fit for the practice of the art, which after all was and must be largely conjectural.

Since then our knowledge of the ancillary sciences has been vastly, in some directions one might almost say infinitely, advanced, yet Celsus's statement remains in the main true.

A working hypothesis was as necessary to the ancients as to us, and their best men groped after one, if haply they might find it. They talked learnedly about temperatures and humours, and found these notions useful in ordering and classifying the knowledge they derived from experience, but beyond that they could not go, nor was any real advance made for about fifty generations, or until the invention of the microscope. That men yearned after something better was evidenced by the discreditable sense which came to be attached to the word empiric.

During the Dark Ages the medical traditions of the Greeks were carried on by Arabian, Persian, and Jewish physicians. We know the names of some of these, as Avicenna, Abenzoar, and Razi; and some of their writings have survived; but of the men we can say little or nothing. Nearly the same thing applies to our own countrymen of that period, though they, like the other European nations, were intellectually inferior to the Orientals; they knew, or thought they knew, a great deal about the properties of drugs and herbs, partly well founded, partly fanciful, and derived from Greek tradition.

As the bright blossom of the Semitic races faded and withered, blasted in the West by the arid blight of Catholicism, and in the East by the substitution of Turkish for Arabian domination, and later by the destructive tide-wave of the Mongol-Tartar flood, intellectual activity sought refuge from the savage and turbulent eddies of Western politics in the quiet, smooth waters of the cloister. We all know the explanation which the apostle of heredity, Francis Galton, found for the lack of progress during the Middle Ages. When inventiveness and love of knowledge were shut up in monasteries and debarred from matrimony, while sturdy stupidity and athletic Philistinism ranged abroad and propagated the species, how could mental development and its fruits make any material advance? There is a well-marked and recognisable difference, generally speaking, between the capacious braincase of a scholar, dug up in the burial-ground of some ruined abbey such as that of the Cistercians recently excavated here in Bristol and the bony, low-browed cranium that we howk up from some forgotten battlefield.

But the absorption of the medical into the clerical profession had the evil effect that might have been anticipated. A blind conservatism took the place of a search for truth; the dogmatic school had too much the upper hand of the empiric, and scholarship of practical skill. The great Linacre, the glory of medicine in the days of Henry VIII., the friend of Erasmus, and the founder of the College of Physicians, was a clergyman; and though he was esteemed as skilful as he was learned, the medical writings he left behind him were all translations from Galen; which is as much as to say that he made no attempt to add to what he considered the true faith.

So strong indeed was this attachment to dogma, that as late as the year 1559 Dr. John Geynes, a man of some mark, was cited before the College for impugning the infallibility of Galen; and only on his humble recantation, signed with his own hand, was he absolved of heresy and received into the College. This, observe, was in 1559. During the latter half of that century several leading members of the College were noted as zealous Catholics. Clearly the scholarly and conservative element still predominated over the scientific, the practical and the progressive. But better times had now arrived, "the spacious times of great Elizabeth;" and medicine was to have its share in the glories and the achievements of the new era.

Christopher Johnson, the accomplished translator of the *Frogs and Mice*, gave up his successful career as head master ot

Winchester School to practise medicine in London, and to write on prophylactics. Penny, a second Dioscorides, as Gerard called him, a clergyman, but—significant fact—a heterodox one, was a really distinguished naturalist. William Gilbert, one of the Queen's physicians, was the founder of magnetism as a science, and the admired of Galileo.

These were the first-fruits of a long succession of men who illustrated their profession by their accomplishments and advanced it by their ability and industry, their imaginative power and their open minds.

On the whole, it would seem that though some who richly deserved success in life never attained to any great measure thereof, yet those who held the front rank in the estimation of their contemporaries were those who most deserved it. They were usually men of learning and travelled men; it seems to me that in somewhat excessive proportion they were the sons of successful apothecaries, of physicians or of dissenting ministers, as senior wranglers are nowadays; in a great many cases they were men distinguished in science and literature outside their profession — as botanists, antiquarians, chemists, natural philosophers, scholars, historians, founders or fellows of the Royal Society. But they were also men of high character and generous disposition, and of affable manners.

The following character-sketch of Dr. William Hunter embraces most of the mental qualities which both deserve and obtain success:[1]—

"There was something very engaging in his manner and address, and he had such an appearance of attention to his patients as could scarcely fail to conciliate their confidence and esteem. In consultation with his medical brethren he delivered his opinion with diffidence and candour. In familiar conversation he was cheerful and unassuming. All who knew him allowed that he possessed an excellent understanding, great readiness of perception, a good memory and a sound judgment; to these he united uncommon assiduity and precision."

Of Dr. Warren, long and perhaps deservedly the head of his

[1] I am much indebted, in this part of the lecture, to Dr. Munk's Roll of the Royal College of Physicians.

profession, it was said that though his natural temperament was cheerful, it was remarkably flexible and sympathetic, and that no one ever had recourse to his advice as a physician who did not remain desirous of gaining his friendship and enjoying his society.

Of course there were exceptions to the rules I have ventured to lay down. Radcliffe was a bear, but he was a genius of the very first water and rarity; and though genius is proverbially difficult of definition, I think we may say with some confidence that it is apt to be accompanied by a certain degree of contempt for human kind and for the rules of society. His want of learning was probably exaggerated by himself and others. He was a graduate of Oxford; and the mode of disposal of his vast wealth showed his respect for erudition.

William Chambers, again, the leading physician in London sixty years ago, is described as possessing no genius, and no brilliant talent or originality. But he had in an eminent degree wisdom, judgment, that peculiar balance of faculties which enables a man to think soundly and to be a safe adviser and guardian; he had also great energy and industry and indomitable perseverance. He is said to have made clear and concise memoranda of every case that came before him, and to have filled therewith no less than sixty-seven thick quarto volumes, an astounding amount of labour for a man with an enormous practice. If genius were really (as it is not) an immense capacity for taking pains, Dr. Chambers would have been a great genius.

One characteristic of the ideal physician hitherto not touched upon is the contempt of danger in the performance of duty which he shares with the good soldier. Of this striking instances occur in the Roll of the College. The names of men who remained at their posts during frightful epidemics of plague, when many others deserted and fled for safety into the country, are well deserving of commemoration. Thus Thorius and Conyers died; and three young men, Alexander Burnett, Glover and Odard, who had dared to investigate the *post-mortem* appearances in this fell disease. Wharton also, eminent as an anatomist, remained in the city to combat the pest, and happily survived, as did also Barwick and Hedges.

As Thorius, a Belgian by the way, and his fellows sacrificed life to duty and conscience, so did Lettsom the gifts of fortune. Inheriting a large property in the West Indies, consisting of a number of negro slaves, he manumitted the whole of them, though the ideas of the period would have permitted him to retain or sell them without blame. He thus reduced himself to penury; but it is gratifying to know that he subsequently obtained and enjoyed the most lucrative practice in London.

Coming nearer to our own time, or rather to the present time, I am irresistibly led to bethink me of the many eminent men whom I used to know intimately, and on the footing of friendship.

There was Alison, the venerable and venerated philanthropist. I can recollect when age and infirmity, of body but not of mind, made him quit his physicianship and clinical professorship in the Edinburgh Infirmary. An interesting and amiable patient of his was just then struggling doubtfully through an attack of typhoid, but after his departure disappointed our hopes by dying suddenly from an imprudent exertion, undertaken against orders, to save trouble to the nurse. A few weeks afterwards I met Dr. Alison painfully dragging his large but feeble frame up the Infirmary stair. "Dr. Beddoe," he said, "you can save me this labour. I only want to know how Monro has got on." I told him the mournful facts. His countenance fell, and without a word he turned and hobbled down the stair. I never saw him again; I doubt whether he ever again left his house.

Then there were Simpson, Christison and Syme, the three stars of Edinburgh in the middle of the century; there were Jenner, James Clarke, Walshe, Parkes, Gull, John Marshall, and William Roberts. They were men of culture as well as of ability and character; their ability was almost as apparent to the laity as to their brethren; and in most of them intellectual energy found a vent in the cultivation of other sciences or arts, generally those ancillary to medicine, but always in strict subordination thereto; much as the molten lava of Etna or Vesuvius cannot all find its way out through the great crater, but must break out in some subordinate vent on the side of the cone. All these men were amiable and of attractive personality, with perhaps two exceptions, of whom the καλος could not be so confidently asserted as the αγαθος.

But I recall the melting sympathy of Simpson, his Jove-like head and kindly, blue eyes; the manly, commanding, almost regal personality of Christison; the keen, practical intellect of Syme and of Jenner; the extraordinary perfection of the senses, and consequent accuracy and quickness of observation and diagnosis, of Walter Hayle Walshe; the high, æsthetic culture and sweet reasonableness of John Marshall; the knowledge of mankind and the exquisite and kindly tact of Sir James Clarke and Sir William Gull; in little William Roberts the lightning quickness of thought, and the warm, genial humour of that "abridgment of all that was pleasant in man"; and in Edmund Alexander Parkes "every virtue under heaven."

It is perhaps hardly admissible to speak of one who is still with us. But I should like at least to mention Lister, whose achievements for the benefit of man have placed his figure in the very centre of the pediment of the great Medical Institute at Rome, and whose benevolent countenance is a true index of his character.

I am not thinking just now of great teachers, but of great and successful practitioners, otherwise Sharpey should be named, big in mind as in body, under whom no pupil could sit and not know his physiology; and Virchow, that magnet of disciples, medical and anthropological; and Paul Broca, whom to know was to love and to labour for, and to think one's self richly rewarded by his approbation.

None of us can attain to our ideal, but to some happy men it is given to approach it, and I think we may number the subject or occasion of this lecture among them. Too indiscriminating a eulogy would not gratify the departed, if he could invisibly listen to it, any more than I should feel happy in uttering it. "Can you not praise the dead man sufficiently unless you tell lies about him?" said Leslie Stephen, best and most veracious critic of men's lives in our time. Surely in this case we can, and if we could not it would be better to keep silence. But in this man I have to do with one who might have said on his deathbed, with the illustrious John Fothergill, that "he hoped he had not lived in vain, but in a degree to answer the end of his creation, by sacrificing interested considerations and his own ease to the good of his fellow creatures."

Without entering into metaphysical subtleties, I think it may be said that we most of us feel that God gave us our intellect, but that we ourselves are responsible for its cultivation and the use we make of it. Edward Fox's intellect was not remarkably brilliant or powerful, but it was clear, healthy, and serviceable; and he devoted himself to its cultivation with a zeal and industry that were an example to us all. Very early in his career I recollect his saying to me, "It is the object of my life to become as good a physician as I can," and well and thoroughly did he carry out that intention, neglecting no subject that belonged to his department, but making some, such as the pathology of the nervous system, peculiarly his own.

Knowledge is power and riches are power; but a knowledge of medicine, such as he built up and retained, is the power of doing good, and often carries with it, as it did in his case, the will to do it, even at great cost of time and labour and thought. It is a trite saying that the healer, the leech, the mediciner—I put aside the word doctor advisedly—is a philanthropist by profession; and he must be a bad man indeed whom the constant practice of so beneficent a profession does not make better. A great and good lawyer, Matthew Davenport Hill, long one of the social ornaments of Bristol, told me he envied me my profession, for, said he, "a medical education tends to make a man better, but a legal one tends to make him worse."

And Fox was a generous man. He was never rich; but even if he had been rich, if he had had that drawback to chill and narrow his spirit, I am sure he would still have been generous, for his heart was large and his soul was warm and sympathetic. He early gave hostages to fortune, but the influence of his domestic circle could but strengthen and foster his generous instincts. During some years he had an uphill race to run: ahead of him were Symonds, the most accomplished and learned of physicians, and Budd, the brilliant if erratic and not always appreciated genius; and he had besides some formidable competitors; but his house was always one to which other strugglers might confidently turn for help and encouragement.

A few years after he had begun practice a serious epidemic of typhus fever broke out in the lower parts of East Bristol. There was then no organisation ready to meet and cope with such a calamity: we had no Medical Officer of Health to say to it, "Thus far shalt thou go and no farther." The better educated people in the slums—nurses and Bible readers and the like—stayed at their posts, but the infection was very fatal among them—brains are a disadvantage in true typhus. The situation appealed to Edward Fox's benevolence and his medical instincts; he created an organisation to meet it, and devoted himself personally to the work. I believe his growing but still small practice suffered considerably, which he could ill afford, but the plague was stayed, and doubtless many lives were preserved.

Fox was truly sympathetic. The quality is an aid to success in practice in more senses than one, but if it sharpens the weapon it corrodes the scabbard. Symonds, who hid a warm heart under a somewhat cold exterior, told me once that though he devoted his mind completely to every case until he had done with it, he made it a rule to dismiss it from his thoughts as soon as he reascended his carriage. A wise rule, no doubt, with his enormous consulting practice; but I do not think Fox could have done likewise. Sitting by his bedside during one of his fits of gout, I was discussing with him the possible exciting causes of the attack. "But after all, Beddoe," he said, "there is nothing to bring on a fit of the gout like a run of anxious cases." It was a cruel inheritance which dogged him through life, and ultimately dragged him down: for he was always a temperate and abstemious man in his own habits, and latterly was an eloquent and forcible preacher of those virtues to others, both on scientific and on moral grounds. In fact, the cause of temperance, the anti-alcoholic movement, has lost in him one of its most powerful champions, at a time when his services were needed almost as much as ever; a champion all the more powerful perhaps because he was no fanatic, and though himself a total abstainer for very many years, was always in perfect charity with those who could not see as he did.

Another cause which he had very much at heart was that of medical missionary enterprise, which naturally commended itself to a man like him, altruistic, truly and spiritually religious, and at the same time zealous for the extension of the benefits of enlightened medical treatment to the ends of the earth. Nor did he neglect the general interests of the profession at home, but for many years devoted much of his scanty leisure to the work of those valuable societies which strive not only to advance medical education and progress, but to maintain a healthy social and ethical tone among their members and in the whole professional body.

His great objects in life were, then, to become a thoroughly good physician, and in that capacity and in such other ways as seemed possible to serve and benefit human kind. And he had the reward which success in such aspirations seldom fails to bring, "Love, honour, troops of friends."

Love and honour will remain attached to his memory: alas! that it is only a memory, and that he himself was not permitted longer to enjoy them.

